# The Late Orchid Catches the Bee: Frost Damage and Pollination Success in the Face of Global Warming in a European Terrestrial Orchid

**DOI:** 10.1002/ece3.70729

**Published:** 2025-01-16

**Authors:** Florian P. Schiestl, Beat A. Wartmann, Ruth Bänziger, Brigitte Györög‐Kobi, Klaus Hess, Jürg Luder, Edith Merz, Beat Peter, Max Reutlinger, Tobias Richter, Heinz Senn, Thomas Ulrich, Beate Waldeck, Claudia Wartmann, Roland Wüest, Walter Wüest, Quint Rusman

**Affiliations:** ^1^ Department of Systematic and Evolutionary Botany University of Zürich Zürich Switzerland; ^2^ Arbeitsgruppe Einheimische Orchideen Schweiz (AGEO) c/o President Beat A. Wartmann Oberengstringen Switzerland

**Keywords:** *Andrena combinata*, climate change, flowering time, frost damage, *Ophrys araneola*, pollination

## Abstract

Global warming changes flowering times of many plant species, with potential impacts on frost damage and their synchronization with pollinator activity. These effects can have severe impacts on plant fitness, yet we know little about how frequently they occur and the extent of damage they cause. We addressed this topic in a thermophilic orchid with a highly specific pollination mechanism, the Small Spider Orchid, *Ophrys araneola* RchB, in six populations in Northern Switzerland. We measured flowering time, frost damage, and fruiting success in 1250 individually marked plants during 3 years, and documented spring temperatures. Using regression models with historical climate data, we estimated past and future frost damage. In addition, we analyzed historical records of the orchid and its only verified pollinator, the solitary bee *Andrena combinata* in Northern Switzerland, to estimate potential desynchronization between flowering and pollinator activity due to climate change. Increased spring temperatures accelerated flowering time, and together with the number of frost days explained frost damage well. Frost damage was severe and early‐flowering plants were more likely to be damaged. Historical climate data suggested frost damage has increased in the last decades and may increase further in the future. All populations but one had very low fruit set, and plants that flowered earlier were less likely to set fruit. The historical data from between 1970 and 2019 showed a significant advance of flowering‐ and pollinator occurrence time in the last decades, but to a similar degree in orchids and bees. Our study shows that the orchid, despite being limited to warm habitats in central Europe, suffers under global warming by increased frost damage caused by earlier flowering. We did not detect an effect of accelerated flowering on desynchronization in flowering time and pollinator activity in this orchid species.

## Graphical abstract

This manuscript documents the effects of flowering time on frost damage and pollination success in wild populations of a European native orchid. It shows that later‐flowering individuals have higher chances of pollination success and lower frost damage.

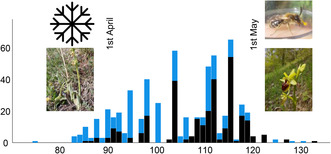

## 1 Introduction

Overwhelming evidence shows that global warming impacts the phenology of plants and animals, with spring‐flowering plants being particularly affected (Fitter and Fitter 2002; Menzel et al. 2006; Cleland et al. 2007; Molnar et al. 2012). These plants have advanced in flowering in the last decades by as much as 10 days (Bartomeus et al. 2011; Robbirt et al. 2014). Such phenological shifts can have diverse effects on plant fitness, because flowering must overlap with the emergence time of the pollinators, and favorable growing conditions to ensure successful seed maturation (Elzinga et al. 2007). Thus, shifts in flowering time can change mutualistic or antagonistic interactions (Tylianakis et al. 2008; Freimuth et al. 2022), plant community structure or invasions (Piao et al. 2019), and damage through abiotic factors such as frost events (Liu et al. 2018).

An important environmental factor affecting reproductive success in plants is the occasion of frost during the onset of the vegetation period or flowering, causing damage to sensitive plant tissue (Cannell and Smith 1986; Thomson 2010). As dates of last frost advances with global warming, the likelihood of frost damage may decrease, however, depending on the advance of flowering time (Scheifinger et al. 2003). A reduced risk of frost damage was indeed predicted by analyses of historical herbarium records across North America and changed occurrence of frost (Park, Ramirez‐Parada, and Mazer 2021). Nevertheless, global warming may also increase frost damage, namely when warm spring temperatures accelerate flowering yet damaging frost events still persist, as exemplified by the devastating frost damage in spring 2007 in the Eastern US (Gu et al. 2008). The phenomenon of increased frost events has been mostly investigated in trees (Cannell and Smith 1986; Augspurger 2013; Wang et al. 2014; Richardson et al. 2018), and alpine herbs, where earlier snowmelt may trigger earlier flowering and a higher exposure of buds and flowers to spring frost events (Inouye 2008). Frost damage also interacts with pollinator attraction, where it may cause reduced pollinator attraction and lower seed set as a consequence (Pardee, Inouye, and Irwin 2018; Pardee et al. 2019).

An indirect effect of phenological changes in plants is the potential desynchronization between flowering and the activity times of pollinators (Renner and Zohner 2018; Freimuth et al. 2022). Whereas for plants with generalized pollination systems little desynchronization (Bartomeus et al. 2011; Forrest and Thomson 2011; Forrest 2015; Razanajatovo et al. 2018) or even increased synchronization has been found (Freimuth et al. 2022), plants with specialized pollination are more likely to suffer from increased mismatch between flowering and pollinator emergence. In the Japanese early‐spring flowering, bumblebee‐pollinated *Corydalis ambigua*, such a mismatch is generated when snowmelt is early but subsequent soil warming is slow, because bumblebee emergence depends on soil warming but flowering is triggered by surface warming after snowmelt. Therefore, plants flower earlier than bees emerge, and seed set is reduced (Thomson 2010; Kudo and Ida 2013; Kehrberger and Holzschuh 2019; Kudo and Cooper 2019). In the terrestrial orchid *Ophrys sphegodes*, the opposite effect has been documented, namely the activity time of specific pollinator bee being more advanced by increased spring temperatures than flowering of the orchid, leading to desynchronization of activity times (Robbirt et al. 2014).

Apart from few case studies, we know very little about how common desynchronization is, despite specialized pollination being relatively frequent among angiosperms (Johnson and Steiner 2000). Also, because studies that investigate desynchronization usually do not document frost damage and vice versa, we know little about the relative importance of these two factors in the face of phenology changes. Lastly, quantification of both effects over multiple years has not yet been done, leading to a gap in our understanding how severe the fitness losses caused by both effects are.

We addressed this gap by a 3‐year field study in six natural populations of a native orchid with a single pollinator species in Northern Switzerland, to quantify the impact of temperature and temperature change on flowering time, inflorescence frost damage and pollination success. We measured frost damage and estimated frost damage in the past and future, using regression models and historical temperature records. To study desynchronization between plant flowering and pollinator activity, we analyzed historical occurrence data of the orchid and its pollinator bee in the region of the study area. Using the multi‐year data of individuals, we also addressed questions about the costs of frost damage and fruiting. We addressed the following specific questions in our study: (1) Is frost damage in this orchid associated to the time of flowering, and how is flowering time determined by spring temperature? (2) What are the likely patterns of frost damage in the past and future given temperature changes in the study region? (3) Is fruiting success in the orchid associated to flowering time? (4) Did the flowering time of *Ophrys araneola* and the emergence time of *Andrena combinata* change differently in the last decades in Switzerland, leading to increased desynchronization between orchid and its pollinator? (5) Does frost damage and fruiting affect the likelihood of re‐flowering in the next year in this orchid species?

## 2 Materials and Methods

### 2.1 Study System

In our study, we investigated the European terrestrial orchid, *O. araneola* RchB. and its only verified pollinator, the solitary bee *A. combinata* (Christ 1791) (Schiestl and Vereecken 2008). This orchid is sometimes treated as a subspecies of *Ophrys sphegodes*, as *Ophrys sphegodes* subsp. *araneola* (Rchb.) M.Laínz, 1983. Like almost all species of the genus *Ophrys*, *O. araneola* is fully outcrossing and employs sexual mimicry for pollination, by imitating the mating signals of female bees and thus attracting males that attempt to copulate with the flower labellum (Johnson and Schiestl 2016). Pollination by sexual mimicry is often species‐specific, and floral scent has been shown to be a key trait for pollinator attraction, with often species‐specific composition, fine‐tuned for the attraction of one or a few pollinator species (Schiestl 2005; Dötterl and Vereecken 2010; Ayasse, Stoekl, and Francke 2011). The genus *Ophrys* is limited to Europe and its adjacent regions and has a primarily Mediterranean distribution. In Central Europe, *O. araneola* is limited to warm‐dry habitats, such as southerly facing nutrient‐poor meadows or open pine forests on limestone soils.

### 2.2 Flowering Time and Temperature in Contemporary Populations

**FIGURE 1:**
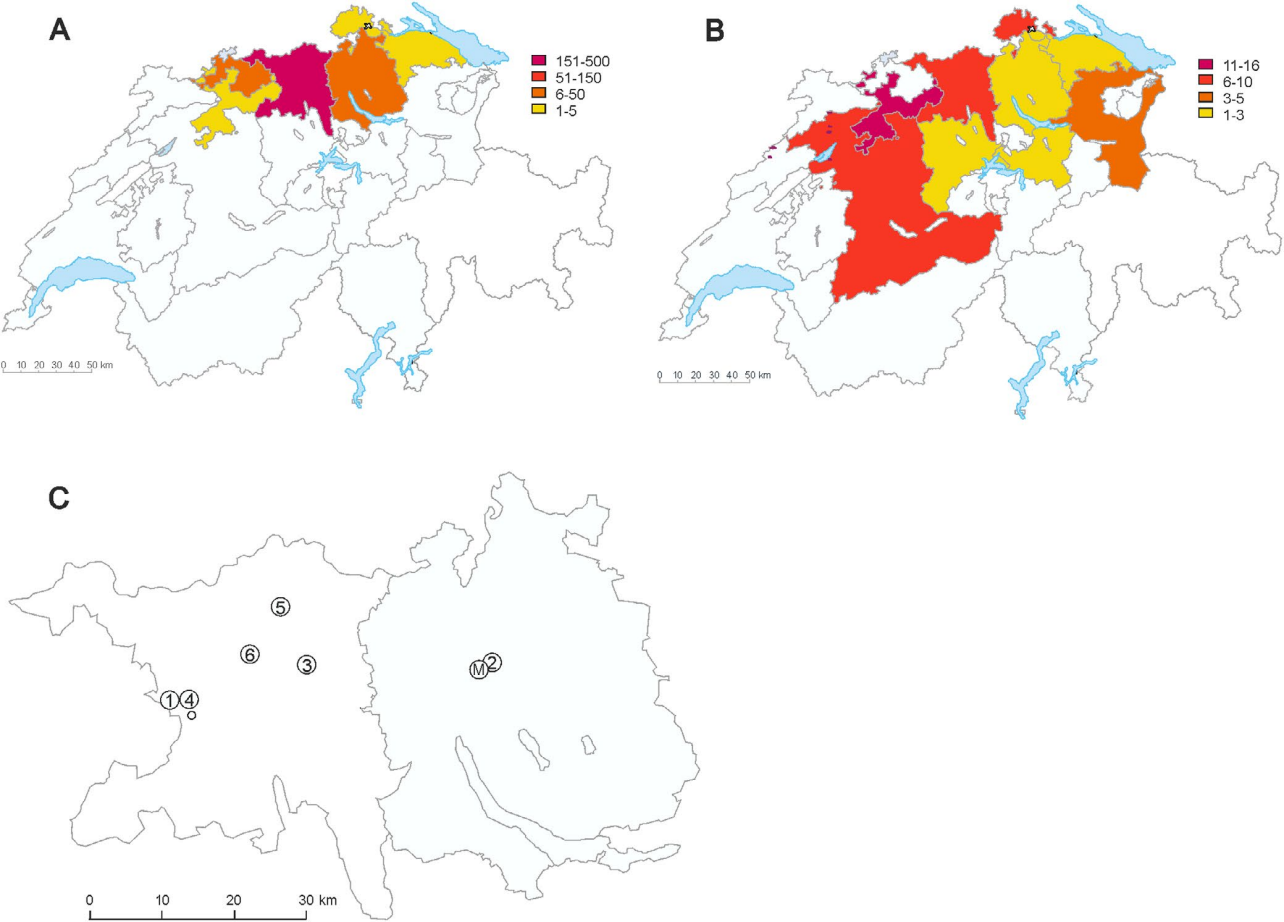
Maps of Switzerland (A, B) and the Swiss cantons Aargau (left) and Zürich (right; C). In A and B, the origin of the historical records of orchids (A) and the pollinator bee (B) used for the desynchronization analysis are indicated, with the color codes referring to the number of records from the respective cantons. Panel C shows the study populations as numbers (for names, see Table 1). “M” indicates the MeteoSwiss climate station at Kloten. Modified from maps provided by the Swiss Federal Statistical Office.

In February 2021, between 100 and 300 rosettes (1250 in total) of *O. araneola* in 6 populations in the Swiss cantons of Aargau and Zürich (ca. 5–30 km apart; Table 1, Figure 1C), where possible close to existing tracks in the field to avoid trampling on plants, were marked individually with metal tags. In some populations, newly discovered plants were marked in 2022 and 2023. Because not every plant flowers every year, and many plants were damaged, especially by frost events, and could not complete flowering, the total number of records obtained from plants throughout the three study years was < 3 × 1250 (i.e., less than all the marked plants in all years). One data logger (HOBO Pendant Temp/Light data logger, model UA‐002‐64) was placed in each population, close to where *O. araneola* plants were growing, to record hourly temperatures. To avoid losing loggers when populations were mowed during late summer/autumn, data logger were removed after flowering, and placed again into the field at the end of the year. All metal tags and data loggers were removed from the field in summer/autumn 2023.

**TABLE 1:** *Ophrys araneola* populations surveyed in this study.

Population	Swiss grid, X‐coordinate	Swiss grid, Y‐coordinate	Latitude	Longitude	Altitude (m)
1 Erlinsbach (Lehrpfad)	2,642,920	1,251,590	47°24′49.40″	8°0′26.39″	530
2 Kloten (Feek, Eigental)	2,689,260	1,258,640	47°28′20.91″	8°37′21.71″	540
3 Birmenstorf (Schluu)	2,661,610	1,257,460	47°27′54.12″	8°15′20.79″	460
4 Küttigen (Judenhalde)	2,646,320	1,252,660	47°25′23.22″	8°3′8.97″	470
5 Villigen (Cheestel)	2,658,810	1,265,670	47°32′20.85″	8°13′11.01″	370
6 Villnachern (Bahnbord)	2,654,420	1,258,420	47°28′27.48″	8°9′37.93″	410

*Note:* All populations are found on southerly facing slopes, with an altitude of 370–540 m above sea level, calcareous soil and semi‐dry meadow plant communities; the populations are annually mowed late in September allowing maturation of orchid seeds.

### 2.3 Flowering Time, Frost Damage, and Fruiting Success

Subsequently, for 3 years, surveys of flowering time, frost damage, and fruiting success in the populations were conducted. Surveys were done every 2–3 days from early March to end of May, where start of flowering of marked plants was recorded. Plants without inflorescence in any particular year were noted as not flowering. Frost damage was assessed during the surveys by recording plants with wilted inflorescences that showed no signs of snail‐ or mechanical damage (Figure S2). We counted only frost damage that totally destroyed inflorescences in any given year, so when frost only partially damaged an inflorescence and the plant continued to flower, this was not counted as frost damage. Thus, our record of frost damage included only plants where the inflorescence had been fully destroyed and subsequently dried out without producing any more flowers in the respective flowering season. Such damage obviously prevented the plant from producing any fruits in the particular season but did not necessarily impact re‐flowering in the next season (see Section 3). After the flowering period (i.e., by mid‐May–June), total number of flowers (wilted, dried flowers are still visible after flowering), number of fruits, and plant height was recorded from all plants that had produced an inflorescence and had not been damaged by frost.

To confirm the presence and identity of pollinators in the populations, we attempted to catch pollinators in all populations by using hand nets and by searching for bees carrying pollinaria on the flowers and in the surrounding of flowering *O. araneola* plants. All researchers doing plant surveys looked for pollinator bees on the flowers; additionally, in 2021 and 2022 several days in the field were devoted by one person specifically for pollinator catching. In 2021, 3 days were spent in Villnachern, and in 2022 4 days in Villnachern and 2 days in Birmenstorf (4 h/day were spent with searching for pollinators on each day). The strategies to find pollinators included sitting in front of patches of orchids for around 30 min to observe visitors, walking around and checking for bees carrying pollinaria. These two actions were alternated during the 4 h. Three male bees were caught in the Birmenstorf population, with two of them carrying pollinaria of bright yellow color typical for the genus *Ophrys*. The bees were identified using a reference specimen of *A. combinata* and the key provided in Amiet et al. (2010). One pollinarium was sequenced using ITS2 primers with standard PCR and Sanger sequencing methods (Breitkopf et al. 2015). The sequence was compared to the DNA extracted from an *O. araneola* leaf.

### 2.4 Historical Orchid/Bee Records/Temperature Data

Historical records of *O. araneola* in Switzerland were obtained from the database “InfoFlora” (www.infoflora.ch). These included records from herbaria dating back to 1868, as well as more recent records collected by professionals and plant enthusiasts in the field. *Ophrys araneola* is sometimes considered a subspecies of *O. sphegodes*, and historically (i.e., before 1950; Schmid 1998) has not always been treated as a distinct species. Therefore, our historical data may contain some *O. sphegodes* records, but as *O. sphegodes* is much rarer than *O. araneola* in the area considered for our study (www.infoflora.ch), the historical dataset of *O. araneola* can be considered reasonably accurate. Very early records up till end of February were deleted, as they can be considered to represent rosettes of individuals not yet flowering. Naturally, the database contains records of plants in different stages of flowering; however, there should be no consistent bias across the years; historical data was not compared to data collected in this study in the field, that focused on the start of flowering, to avoid issues with different stages of flowering in the datasets. Records of the pollinator bee, *A. combinata* (males and females) were obtained from the Swiss database “InfoSpecies” (Swiss National Apoidea Databank https://doi.org/10.15468/ksfmzj); as for the orchid data, these data comprise specimen of insect collections and records from bee‐collectors, going back to the 1970s. The data did not indicate the gender of the bees (only male bees are pollinators of *Ophrys*), making a male‐specific analysis impossible; although in several bee species, males emerge before females, this seems to be not be the case for *A. combinata* (Westrich 1989; Schiestl and Vereecken 2008), making a combined analysis of males and females reasonable. For the analysis of flowering advancement, all orchid data (1868–2019) were used. To analyze desynchronization, only the time period for which orchid and bee data were available (i.e., from 1970 to 2019) was used. In addition, to achieve a sample from a climatically and topographically homogeneous region, we only used data from Northern Switzerland (comprising the greater region where the field work was conducted, excluding the north‐western region). Specifically, 52 bee records and 725 orchid records from the cantons of St. Gallen, Thurgau, Schaffhausen, Zürich, Aargau, Luzern, Schwyz, Solothurn, Basel‐Land, and Bern were used (Figure 1A,B).

Historical data of number of frost days in April (i.e., days with temperature < 0°C) and mean temperature in April was obtained from the years 1950 to 2023 from MeteoSwiss for the recording station “Kloten”, which had the furthest back reaching continuous data records available for Switzerland, and was close (ca. 3 km) to one of our orchid populations (indicated as “M” in Figure 1C).

### 2.5 Statistical Analysis

#### 2.5.1 Data of Population Surveys: Temperature and Flowering Time

Generally for flowering time/bee emergence, all date‐values were converted into “day of the year” to allow statistical analysis of flowering time as an ordinal explanatory variable. Temperature data obtained from the data logger were used to calculate mean temperatures and number of days with frost (< 0°C) for the months January–June for each population and study year. For the rest of the months, not enough data were available to allow a robust analysis. A maximum of 18 data points per month could be gained (6 populations and 3 years); however, because data loggers were lost in the field or ran out of battery power, all 18 data points were not collected across all months. Pearson product moment correlations were then calculated between mean temperatures of the 6 months that were analyzed and the mean “start of flowering” and “earliest flowering time” in the populations. We initially tested different regression models with mean temperature and number of frost days of the different months, to predict frost damage. Pearson product moment correlations were also calculated for the measured traits of individuals between pairs of years (i.e., “start of flowering”, “number of flowers”, “plant size”). Spearman rank correlations were used for binary (frost damage, flowering) and count variables (number of fruits).

#### 2.5.2 Frost Damage

Factors impacting the degree of frost damage in orchid populations were assessed by a generalized linear mixed model (GLMM), with frost damage as the (binary) response variable (no‐damage/damage). “Population” and “year” were included as random factors, “day of first flower” as covariate, and the interaction between “day of first flower” and “population”, and “day of first flower” and “year”. In addition, the association between flowering time and frost damage was estimated as using binary logistic regression, for each population and year separately, to allow for the estimation of population‐ and year‐specific regression coefficients (that can be seen as an estimation of selection coefficients). In total, 16 coefficients (out of 18 year/population combinations) were calculated, because in two populations, no frost damage was recorded in 2023.

To estimate historical frost damage reaching back to the year 1950, the regression model obtained from the frost damage and temperature data collected with the dataloggers from all our study population was applied to the historical temperature data from “Kloten”. Our regression analyses showed that mean temperature in April and number of frost days in April explained best the variance in frost damage in the contemporary data (see Section 3) and was therefore considered suitable for the estimation of past frost damage. The model yielded the regression equation “frost damage” = (5.59 × “number of frost days in April”) + (6.25 × “mean temperature in April”) − 79, that was used to estimate frost damage. For the prediction of frost damage in the future, until the year 2050, data for mean temperature and number of frost days in April were created using statistical parameters in the historical data. First, mean temperatures in April were estimated using the regression equation derived from the historical temperature data from Kloten, which predicted a significant increase in mean April temperature: “mean temp in April” = (0.03 × “year”) − 51.41 (*R* ^2^ = 0.16, *p* < 0.001). To account for the variation around the mean, we created random numbers with the mean values derived from the regression model for every year and a constant standard deviation of 1.6 (calculated from the historical data, assuming variation in spring temperature will not change). To produce data on number of frost days in April, random numbers with a mean of 4.7 and standard deviation of 3.3 were created for each year (negative values were rounded to 0). These mean and SD values were calculated from the historical data and using them to predict future data was considered appropriate as the historical data did not predict any change in the number of frost days in April (regression with year as independent‐ and number of frost days as dependent variable: *R* ^2^ = 0.007, *p* = 0.479). For the predicted climate data, 100 replicates were created, and for each dataset, frost damage was again estimated using the regression model obtained from the contemporary population data. Finally, with the predicted frost damage for the historical data (1950–2023) and the 100 datasets of “future data” (i.e., from 2024 to 2050), linear regressions with “year” as independent‐ and “predicted frost damage” as dependent variable were calculated to assess how often the data would predict a significant increase of frost damage in the future. The relationship between mean temperature in April and number of frost days in April was assessed for three time categories (1: 1950–1985; 2: 1986–2023; 3: 2024–2050) by running a general linear model with predicted frost damage as dependent variable, time category as fixed factor and mean temperature and frost days as covariates, and the interactions between factor and covariates. For the future time category, one of the 100 predictions was randomly chosen.

#### 2.5.3 Fruiting Success

Because few plants had fruits, and the number of fruits was generally low (mean [±SEM] = 0.07 [±0.10]; see Table S3), the numerical fruit set data was transformed into a binary data (no‐fruits/fruits), to avoid problems with many zero values in the analysis. Factors impacting fruit set were assessed by a generalized linear mixed model (GLMM) with binary distribution with “no‐fruits/fruits” as dependent variable. Plants with frost damage were not included in this analysis, as they did not successfully complete flowering. “Population” and “year” were included as factors, “day of first flowering”, “number of flowers” and “plant height” as covariates; the interaction between “day of first flowering” and “population”, and “day of first flowering” and “year” was also included in the model. The association between flowering time and fruit set was also investigated for the population “Birmenstorf” only and for each year separately, as for the other populations, the number of plants with fruits was considered too low for a meaningful analysis (see Table S3).

#### 2.5.4 Analysis of Desynchronization

To assess the flowering time of *O. araneola* throughout the time period of the historical records, the data was sorted according to its year into four time categories, A: 1868–1900, B: 1901–1940, C: 1941–1980, D: 1981–2019, and the mean values for the dates of those records was calculated. To estimate any change in flowering time throughout the last 150 years, a linear regression with “day of the record” as a dependent variable and “year of record” as independent variable was calculated (a quadratic regression was also performed and produced very similar values). In this analysis, a negative slope of the regression line (*β*) indicates an advance in the dates of the records, and a positive slope indicates a delay in the dates. To compare historical bee‐ and orchid data, only data from the same time period (1970‐present) and geographic region were used (north‐eastern Switzerland, the region in which the study was conducted). To analyze difference in the change of orchid flowering and bee occurrence, a general linear model was used with “day of the record” as dependent variable, “species” (i.e., orchid/bee) as fixed factor, “year” as covariate, and the interaction between “species” and “year”. Here, a significant interaction between “species” and “year” indicates a difference in the way “date of the record” and “year” are associated with each other in orchids and bees. This would indicate a difference in the way orchid flowering and bee emergence changes throughout time. To compare flowering time and bee emergence, a one‐way ANOVA with “date of the record” as dependent and “species” as factor was conducted. To analyze the relationship between spring temperature desynchronization of orchid flowering and bee emergence, the correlation between temperature in April (data from Kloten), mean date per year of orchid records and bee emergence, and the difference between the two (representing a value for desynchronization) was calculated for the data between 1970 and 2019. All statistical analyses were performed with IBM SPSS statistics version 14.

## 3 Results

### 3.1 Data of Population Surveys

In our study populations, mean start of flowering varied considerably, from 25th March (day 84) in Villnachern in 2022, to 25th April (day 115) in Kloten in 2021 (Table 2, Table S3). Start of flowering of the same individuals was correlated highly significantly among the years: 2021–2022: *r*_372_ = 0.84, 2021–2023: *r*_342_ = 0.71, 2022–2023: *r*_402_ = 0.67, all *p* < 0.001. The same was found for “number of flowers” and “plant size”, where values of individuals between years were highly significantly correlated (number of flowers, 2021–2022: *r*_224_ = 0.38; 2021–2023: *r*_237_ = 0.40, 2022–2023: *r*_302_ = 0.39 all *p* < 0.001; plant size, 2021–2022: *r*_120_ = 0.46, 2021–2023: *r*_101_ = 0.40, 2022.2023: *r*_233_ = 0.40, all *p* < 0.001), indicating constant environments and/or heritability of these traits. Mean temperature in February showed the strongest correlation with mean start of flowering, suggesting earlier flowering with higher temperatures in February (Table 2). This association was also highly significant for “earliest flowering”; the association between mean temperatures and flowering time was similar for the other months (yet not significant).

**TABLE 2:** Correlation between flowering time and average temperatures in January–June in the six populations, assessed within 3 years.

Mean temperature per month (no. data points)	Mean start of flowering	Earliest flowering
January (9)	−0.48 (0.192)	−0.25 (0.518)
February (10)	**−0.70** (0.024)	**−0.656** (0.040)
March (13)	−0.03 (0.930)	0.07 (0.815)
April (16)	−0.42 (0.105)	−0.40 (0.125)
May (16)	**−0.50** (0.046)	**−0.52** (0.040)
June (10)	−0.21 (0.570)	−0.51 (0.133)

*Note:* Values show Pearson product–moment correlation coefficients (*r*), with *p* values in parentheses. Significant values are shown in bold. Numbers behind months are number of data points used in the analysis.

### 3.2 Frost Damage

**FIGURE 2:**
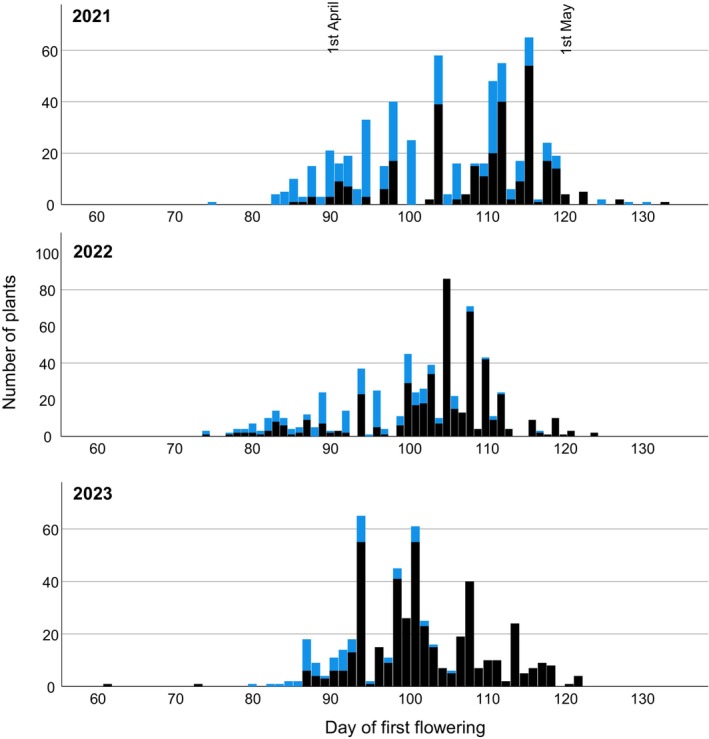
Flowering time and frost damage in plants in all six study populations, shown separately for the three study years. Plants suffering frost damage are indicated as the top, light blue section of the bars. The figure shows that frost damaged decreased with later flowering throughout all three years, albeit with different levels of significance (for statistical values see Tables 3 and 4).

Frost damage was variable and reached high levels with over 80% of plants damaged and unable to complete flowering in some populations and years (Figure 2). Frost damage of individuals was correlated among years (2021–2022: *ρ*_400_ = 0.37; 2021–2023: *ρ*_349_ = 0.29; 2022–2023: *ρ*_352_ = 0.19, all *p* < 0.001), indicating many individuals were damaged in multiple years. Frost damage in 2021 had no effect on flowering of individual plants in 2022 (*ρ*_541_ = −0.05, *p* = 0.210), but frost damage in 2022 had a significant negative effect on the likelihood of flowering in 2023 (*ρ*_510_ = −0.13, *p* = 0.005).

Our analysis showed that “day of first flower” (i.e., flowering time) highly significantly explained frost damage; as expected, early‐flowering individuals were much more likely to be damaged by frost (Table 3; Figure 2). “Population”, “year”, and the interaction between “year” × “day of first flower” were also significant in the analysis, suggesting frost damage varied especially among years and the impact of flowering time on frost damage varied among years. In the analysis with years and populations separately, 12 out of 16 year/population combinations showed a significant association between flowering time and frost damage (Table S1; 2 populations in 2023 had no frost damage).

**TABLE 3:** Factors impacting frost damage in the study populations assessed by a generalized linear mixed model with binomial distribution.

Source	*χ* ^2^	df	*p*
**Population**	**11.55**	**5**	**0.041**
**Year**	**17.08**	**2**	**< 0.001**
**Day of first flower**	**114.17**	**1**	**< 0.001**
Population × Day of first flower	7.61	5	0.179
**Year × Day of first flower**	**25.64**	**2**	**< 0.001**

*Note:* Frost damage (no‐damage/damage) was used as dependent variable, “population” and “year” as factors, and “day of first flower” as covariate. Interactions between factors and the covariate were included, too. Besides population and year, flowering time had a highly significant impact on frost damage, with early‐flowering plants being more affected than later‐flowering ones (significant values are given in bold; see also Figure 4). A total of 1978 plant records, based on 1147 individually marked plants were included in this analysis (some plants were assessed during more than 1 year).

For the temperature parameters, a linear multiple regression with “mean temperature in April” and “number of frost days in April” as independent variables was highly significant in explaining percent frost damage (*R* ^2^ = 0.85; *p* < 0.001). Of all the temperature/frost days combinations, these two parameters yielded the model with the highest *R* ^2^ value, except a model with mean temperatures of Jan‐Apr and no. of frost days in April that had a slightly higher *R* ^2^ value but was not significant (*R* ^2^ = 0.91, *p* = 0.09).

### 3.3 Estimating Historical and Future Frost Damage

**FIGURE 3:**
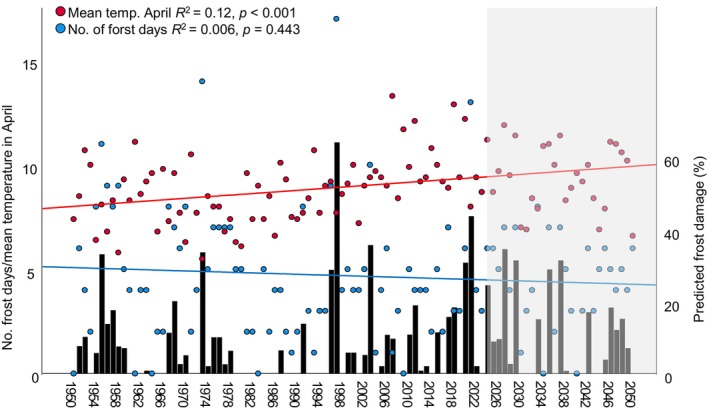
Mean temperature and number of frost days in April (red/blue dots, measured in “Kloten” from 1950 to 2023 and predicted through regression‐based models [see Section 2] for 2024–2050), and estimated frost damage (black bars). The gray area indicates the future‐prediction data; for this time period, 100 estimations were done and one estimation was randomly picked for the figure. For the majority of the 100 estimations, our models predicted an increase in frost damage from 1950 to 2050 (see Section 3).

**FIGURE 4:**
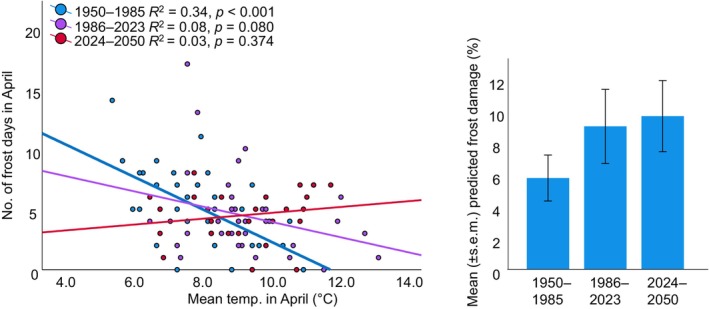
Relationship between mean temperature and number of frost days in April, across the three time categories (1: 1950–1985; 2: 1986–2023; 3: 2024–2050). Only in the first time category this association is significant. Right: Change of percent frost damage across the three time categories (for statistical values, see Table S2). For time category 3, the same randomly picked data dataset as shown in Figure 3 was used.

Our estimation of historical frost damage is based on a linear multiple regression model with “mean temperature in April” and “number of frost days in April” as independent variables, that explained 85% of variance in frost damage in our populations (see Section 2). With the past temperature records from Kloten, the model predicted well the percent frost damage in this population for 2021 (predicted: 44%; real: 31%) and 2022 (predicted: 2%; real: 1%) but underestimated frost damage for 2023 (predicted: 0%; real: 15%). The estimated historical frost damage suggested severe frost damage in several years (1950s, 1970s), followed by many years without any damage (Figure 3). From 1996 onwards, damage seems to have become more regular. Of the two parameters that determine frost damage, namely mean temperature and number of frost days in April, the historical climate data show significant increase in mean temperature, whereas the number of frost days stayed the same (Figure 3). The strong negative correlation between mean temperature and number of frost days in April in our time category 1 (1950–1985) disappeared in the time category two (1986–2023) and three (2024–2050; Figure 4). Our regression models including the estimated future temperature parameters predicted significant increase in frost damage in 59 out of 100 replicates (*p* < 0.05), in eight replicates *p* values were > 005 and < 0.06, and in 33 replicates *p* values were > 0.06. Discarding the eight marginally significant cases, this shows a significantly more frequent prediction of increased damage ($\chi_{1}^{2}$ = 7.35, *p* = 0.007). Our analysis of predicted frost damage showed significant increase in damage in the more recent time categories (Table S2; Figure 4).

### 3.4 Fruiting Success

**FIGURE 5:**
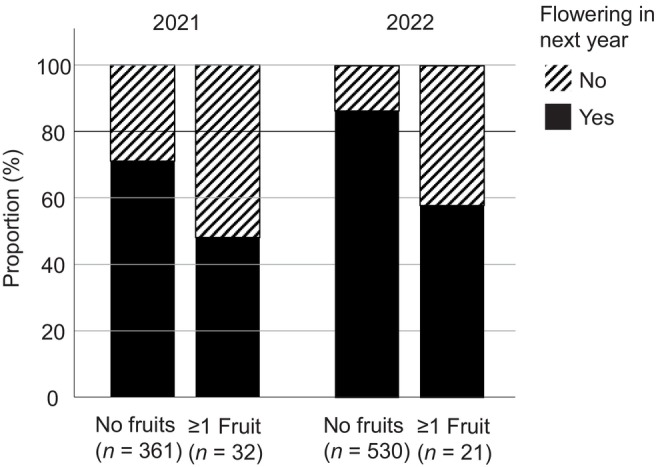
Plants that produced one or more fruits had a reduced likelihood of flowering the next year, indicating a trade‐off between fruiting and subsequent flowering (see Section 3 for statistical values).

Fruit set was low in most populations (Table S3) with the percent of individuals with at least one fruit ranging between 15 (Birmenstorf in 2021) to 0 (plants in Erlinsbach did not produce fruits throughout all study years, except one plant which set 2 fruits in 2022, and two plants that set a total of three fruits in 2023, both outside the study area), with a total mean (±SD) of 3.43 (±4.58) for all populations and years (Table S3). The mean number of fruits produced by all plants of a population varied between 0.32 (±0.73) (Birmenstorf) and 0, with a total mean of 0.07 (±0.10) for all populations and years. Number of fruits produced by individuals was significantly positively correlated between 2021 and 2023 (*r*_238_ = 0.31, *p* < 0.001), and between 2022 and 2023 (*r*_318_ = 0.15, *p* = 0.008). Nevertheless, the number of fruits produced in 1 year had a significant negative effect on the likelihood of flowering in some of the following years (2021–2022: *ρ*_337_ = −0.14, *p* = 0.012; 2021–2023: *ρ*_389_ = −0.05 *p* = 0.390; 2022–2023: *ρ*_362_ = −0.18, *p* < 0.001; Figure 3), and on number of flowers produced in some of the following years (2021–2022: *ρ*_217_ = −0.18, *p* = 0.009; 2021–2023: *ρ*_232_ = −0.17, *p* = 0.008; 2022–2023: *ρ*_301_ = 0.02, *p* = 0.680), suggesting a trade‐off between fruiting and plant performance in the following years (Figure 5).

**FIGURE 6:**
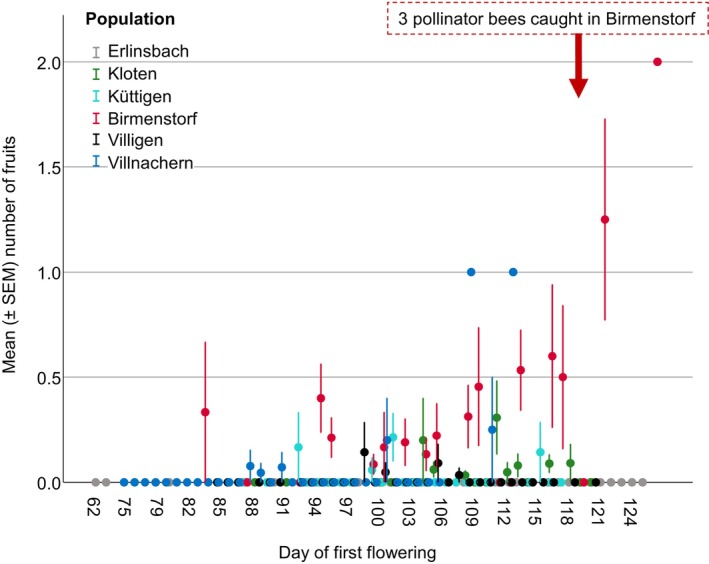
Relationship between mean number of fruits produced by plants in the study populations and their “day of first flowering”. Fruiting success differed between year and population, and was positively associated to plant size, and “day of first flower” in a population‐ and year‐specific way (significant interaction between “days of first flower” and “year”, and “day of first flower” and “population”; for statistical values, see Table 4 and Table S4).

The likelihood of producing at least one fruit varied according to population and year, and was significantly associated with plant size (taller plants had higher likelihood of producing fruits; Table 4). Whereas flowering time was not significant in this analysis including all populations, the interactions between flowering time and year, and flowering time and population were significant suggesting flowering time is an important predictor of fruiting success in some populations and years (Table 4). When the analysis was done for the population with the highest fruit set only (Birmenstorf), the likelihood of producing at least one fruit showed a significant association with flowering time in 2021, with a higher likelihood of producing fruits for later‐flowering individuals (Figures 5 and 6; Table S4).

**TABLE 4:** Factors impacting the number of fruits produced by the plants assessed by a generalized linear mixed model with binary distribution (no‐fruits/fruits), using plants of all populations.

Source	*χ* ^2^	df	*p*
**Population**	**11.93**	**5**	**0.036**
**Year**	**12.56**	**2**	**0.002**
Day of first flower	0.00	1	1.00
Number of flowers	0.61	1	0.44
**Plant size**	**6.19**	**1**	**0.013**
**Year × Day of first flower**	**13.40**	**2**	**0.001**
**Population × Day of first flower**	**13.19**	**5**	**0.022**

*Note:* “No‐fruits/fruits” was used as dependent variable, “population” and “year” as factors, and “number of flowers”, “plant size” and “day of first flower” as covariates. Significant factors are shown in bold. This analysis was based on a total of 987 individually marked plants (Erlinsbach: 90, Kloten: 330, Küttigen: 114, Birmenstorf: 168, Villigen: 208, Villnachern: 77) that produced a total of 1528 inflorescences that completed flowering without frost damage throughout the three study years.

**FIGURE 7:**
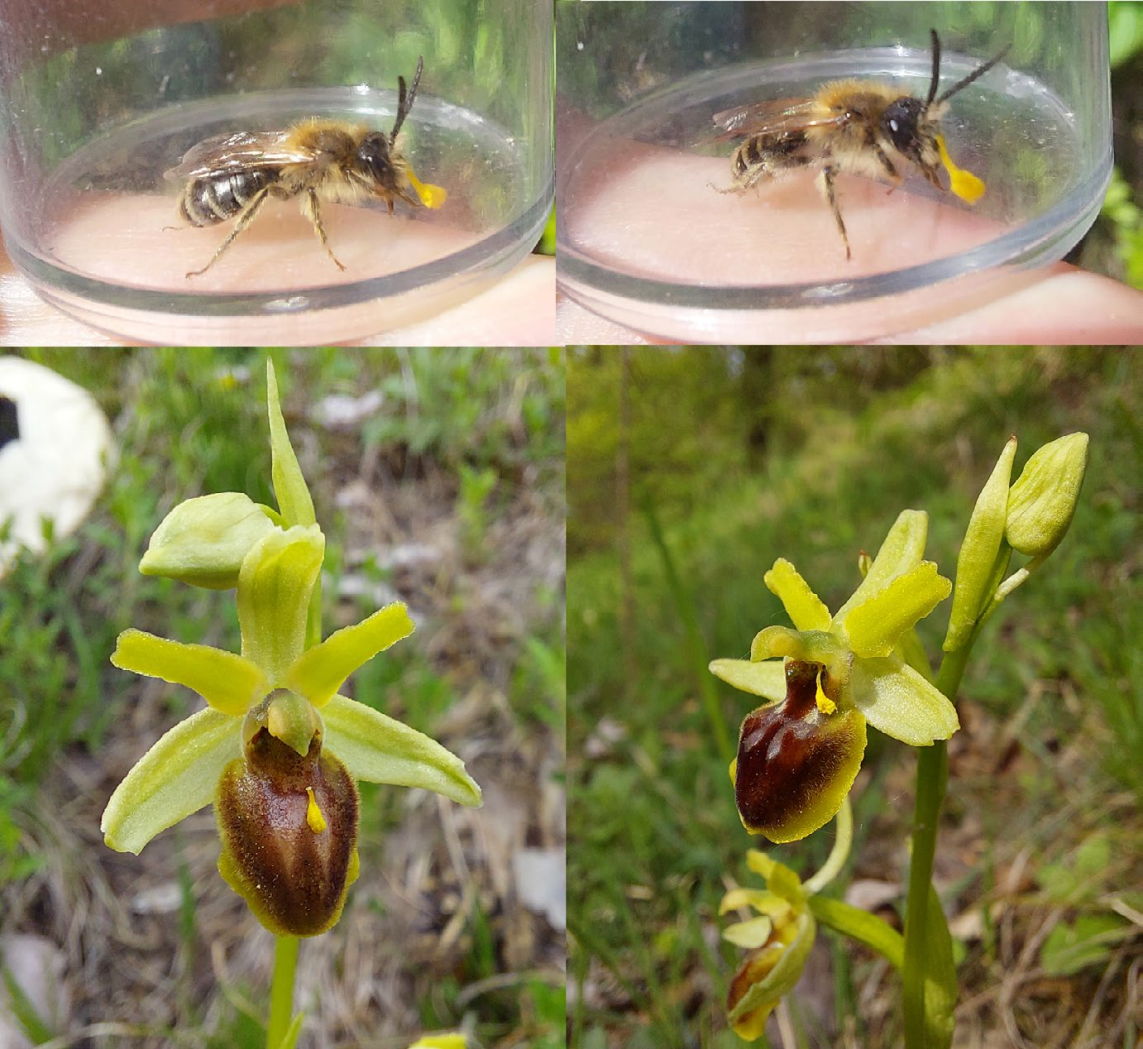
Pictures of one individual male of *Andrena combinata* carrying pollinaria of *Ophrys araneola*. The identity of the pollinaria was confirmed by ITS2 sequences. Below are two flowers of *O. araneola* with one loose pollinarium, apparently deposited by the pollinator bees. Pictures of both bees and orchids were taken on April 29th 2023 in the population Birmenstorf. Pictures by Quint Rusman.

Three males of *A. combinata* were caught on 29th April 2023 in the population Birmenstorf (Figure 7). Two of the bees carried pollinaria, the ITS2 sequence of which were identical to the one derived from an *O. araneola* leaf (Figure S1). ITS2 is not species‐specific in the genus *Ophrys* but differs between *O. araneola* and *O. insectifera* (Bateman et al. 2003), with *O. insectifera* being the only co‐flowering *Ophrys* species during the time of the study in the Birmenstorf population.

### 3.5 Historical Data: Flowering Time and Activity of the Pollinator Bee

**FIGURE 8:**
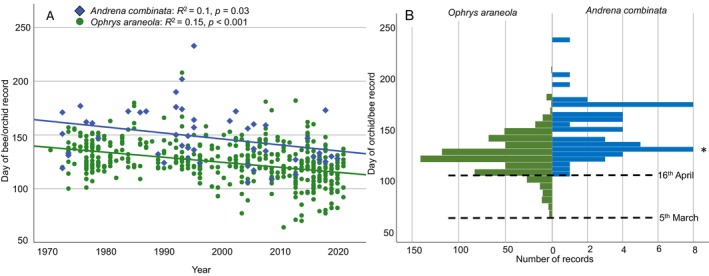
Historical records of *Ophrys araneola* and its pollinator, *Andrena combinata* in Northern Switzerland (data obtained from www.infoflora.ch, www.infospecies.ch). (A) Date of record drawn against the year of the record, showing that both, flowering time of the orchid and the emergence of the bee, have advanced similarly in the last 50 years in Switzerland. (B) Histogram of abundances of records at the different dates, showing the orchid to flower significantly earlier (ca 20 days) than the bees to emerge (mean ± SD, orchid: 123.64 ± 18.28 (3rd May), bee: 147.63 ± 25.46 (27th May); *F*_1,717_ = 77.8, *p* < 0.001); *on 29th April 2023, three males of *A. combinata* were caught at the population Birmenstorf, two of which carried pollinaria of *O. araneola*.

**FIGURE 9:**
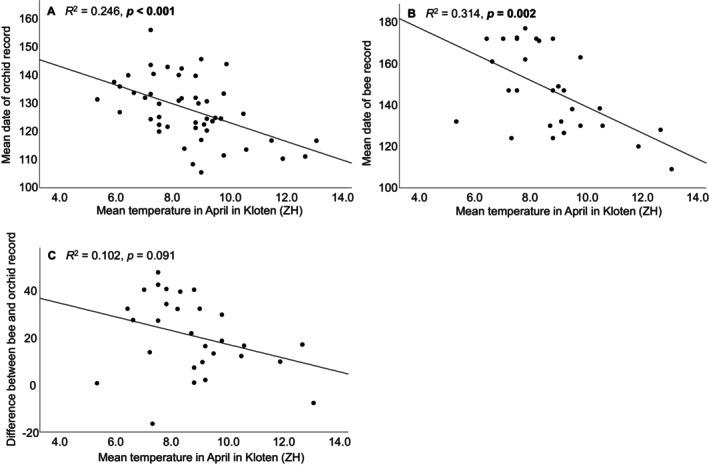
Relationship between flowering of the orchid (A), emergence of the pollinator bee (B) and the difference between the two (C), in relation to mean temperature in April from 1970 to 2019 (measured in Kloten, Kanton Zürich). The analysis shows that warmer temperatures in April triggered earlier flowering and bee emergence and tended to reduce the difference between orchid flowering and bee emergence. This result supports the finding that whereas global warming accelerates flowering and bee emergence, it does not increase the desynchronization between the orchid and its pollinator bee in this region.

Our analysis of historical *O. araneola* records showed that within the last 150 years, the flowering time of this orchid has advanced by about 12 days in Switzerland (Figure 8A shows only the last 50 years, i.e., the time where bee records were available, too). The mean “time of the record” in 4 year‐categories changed from 12th May in the 19th century (1869–1900; day 132 ± 10.42, *n* = 16) to 8th May (1901–1940; day 128 ± 10.2, *n* = 32, and 1941–1980; day 128 ± 16.23, *n* = 164) to 30th April (1981–2019; day 120 ± 17.58, *n* = 836). Using data of all individual years, a highly significant advance in flowering time was detected (*β* = −0.24, *p* < 0.001). The analysis for the time period where both orchid and pollinator‐bee data were available (1970‐present) showed a similar advance of flowering time in orchids and emergence time in bees (bee: *β* = −0.31, *p* = 0.030, orchid: *β* = −0.38, *p* < 0.001; Figure 8A). There was no significant difference in the way flowering time and bee emergence advanced during this time period (interaction species and year: *F*_1_ = 0.25, *p* = 0.620). Comparing the abundances of records throughout the flowering/bee‐activity season showed that orchids flowered consistently earlier than bees emerged, with the earlier flowering orchids well ahead of the first bees (earliest orchid record: 5th March [3rd March in our data], earliest bee record: 16th April [Figure 8B]). The mean flowering time of the orchid was also significantly earlier than emergence time of the bee (*F*_1,717_ = 77.8, *p* < 0.001). Both orchid flowering time and bee emergence showed a significant negative relationship to spring temperature in the years 1970–2019 (Figure 9) showing warmer springs lead to earlier flowering/bee emergence. The difference between mean orchid flowering and bee emergence was not significantly related to spring temperature, but showed a negative trend (Figure 9), indicating higher spring temperatures do not lead to more desynchronization (rather the opposite).

## 4 Discussion

Climate change affects plants by impacting phenology, performance, or biotic interactions, leading to altered fitness and range contractions or expansions as a consequence in many species (Parmesan and Hanley 2015). The orchid we investigated here is confined to warm and dry habitats in Central Europe, having a primarily Mediterranean distribution (Delforge 1995); for pollination it is dependent on a single bee species (Schiestl and Vereecken 2008). Both net positive or negative effects through global warming are thus imaginable in this plant species: it may profit from an expansion of warm habitats in Central Europe, yet suffer under desynchronization with its pollinator (Robbirt et al. 2014). We document unexpected and severe impact of frost in early‐flowering individuals, with flowering having advanced significantly within the last 150 years. Like shown for many other plant species, this is caused by increasing spring temperatures (Piao et al. 2019). Because the number of frost days has remained the same in our study area, the percentage of plants affected by frost damage has increased. For fruit set, we also show that later‐flowering individuals have a higher likelihood of being pollinated by the specific pollinator. Our historical data, supported by our own pollinator observations, suggest that the orchids' flowering and the pollinators' emergence are indeed little synchronized, yet the degree of desynchronization seems not to have changed in the last decades due to climate warming. We thus suggest that global warming affects the orchid negatively, primarily through increased frost damage. Our study system may be representative for plants of Mediterranean origin in Central Europe, that have spring temperature triggered flowering yet low frost tolerance in the flowering shoot. Whether plants can mitigate this effect by adaptively evolving later‐flowering time or increased frost tolerance is an interesting question for future investigations.

The fact that global warming increases frost damage in some plants seems counter‐intuitive but stems from the fact that whereas spring temperatures increase, severe frost events in spring may remain frequent (Cannell and Smith 1986; Gu et al. 2008). The effects of this varies across regions and species (Liu et al. 2018), with some studies suggesting frost damage in trees has increased with global warming (Augspurger 2013), others find no such effects (Scheifinger et al. 2003). In alpine wildflowers, experiments mimicking earlier snowmelt showed that advanced flowering had negative effects through frost damage and pollinator attraction in species with earlier flowering time, whereas those that naturally flower later profited from accelerated flowering (Pardee et al. 2019). Our estimation of past frost damage suggests that frost damage has increased up till now and may further increase given pattern of climate change remain similar. The effect of this on orchid demography is less clear. Although individual plants are not killed by the frost and may re‐flower in the next year, we also document reduced likelihood of re‐flowering after frost damage in one of the two between‐year observation periods. This suggests that frost damage incurs costs for the plants and may be linked to higher mortality. On the other hand, because fruit set seems to be limited to later‐flowering individuals anyway, the impact of frost on the reproduction of the population may be limited.

Our data on desynchronization contrast with earlier work on the closely related *O. sphegodes* and its pollinator bee, *Andrena nigroaenea* , from England, that documented the bee emerging significantly earlier than the flowering of the orchid (Robbirt et al. 2014). In addition, in this orchid‐pollinator association, climate change seems to advance the flight time of the bee more strongly than the flowering time of the orchid, suggesting an increasing desynchronization between them in the future (Robbirt et al. 2014; Hutchings et al. 2018). For *O. sphegodes* in England, Robbirt et al. (2011) calculated a 6d advance in flowering per 1°C temperature increase in spring. In Switzerland, the mean annual temperature has increased by about 2°C in the last 150 years (Appendix S1), and our observed advance in *O. araneola* flowering is 12d, which perfectly matches the estimated advance in *O. sphegodes* flowering. For *A. nigroaenea* , Robbirt et al. (2014) showed an advance in emergence of up to 11.5 d/C, which is about double the value we document for *A. combinata* . Although our dataset with 52 bee records is less extensive than the one used by Robbirt et al. (2014) which comprised 357 bee records, the two bee species may well respond differently to increased temperatures. In bees, species that overwinter as adults tend to emerge earlier with warmer winter temperature, whereas those that overwinter as larvae tending to emerge later (Frund, Zieger, and Tscharntke 2013). Whether the two species, *A. combinata* and *A. nigroaenea* , really respond differently to warmer spring temperature and what the reason for this may be, needs more investigation in the future. Our documentation of the bee pollinator in one of the populations, Birmenstorf, despite being anecdotal, aligns with the peak record of this bee species in N‐Switzerland (Figures 5 and 6) and thus supports the later occurrence of this bee species in relation to the flowering of the orchid. The observation of the pollinator bee in the population with the highest rates of fruit set also supports *A. combinata* as the only (known) pollinator of *O. araneola* (Schiestl and Vereecken 2008), and the assumption that patterns of fruit set in our study populations are caused by natural pollination, rather than undocumented hand pollination by orchid enthusiasts.

Our data of individually marked plants highlighted correlations between several kinds of traits of the same individuals among years. For example, flowering time was highly consistent throughout the years, and the same was true, but to a lesser degree, for plant size and number of flowers produced. As trait variability is caused by environmental‐ as well as genetic factors, consistent traits among years indicate either constant environmental conditions and/or genetic components of the traits. A constant environment is indeed suggested by our finding of correlated frost damage and fruit set in plants among years. Also, a trade‐off between fruiting and subsequent flowering was indicated by our data by the fact that plants with fruits had a lower likelihood of flowering the next year again. Such a trade‐off was already suggested by earlier studies in orchids, albeit in a population‐, climate‐ and species‐specific way (Primack and Hall 1990; Calvo 1993; Sletvold and Ågren 2015a, 2015b), and suggests that fruiting is costly in these orchids. Low fruit set has been found in other populations of *O. araneola* (Claessens and Kleynen 2011) and may represent, in connection with long distance of pollen flow typically found in sexual mimics (Johnson and Schiestl 2016), an adaptation for the production of fewer but high‐quality seeds.

Our here documented frost damage and late emergence of the pollinator suggest that the orchids are under selection for later flowering (see also estimations of selection coefficients in Tables S1 and S4). Thus, it is a puzzling question whether later flowering will evolve in the orchids and why later flowering, leading to less frost damage and a better synchrony with the pollinator bee has not yet evolved. Low heritability of flowering and/or the long generation time in this orchid may slow down the evolutionary response to selection for later flowering. In the future it would be interesting to determine genetic variance and covariance of flowering time in this and other orchid species, for example, by using genomics approaches, to find loci associated to flowering time and estimate the evolvability of this trait. Together with figures of natural selection, this would allow to build models on the speed of adaptive evolution, and thus to predict how fast populations can adapt to global warming. Such information can help to inform conservation measures such as assisted migration or hand pollination. Conservation should prioritize enabling and fostering of natural pollination by the pollinator bee, through conserving bee habitats and attempting to re‐establish pollinator‐bee populations where possible. As a last opportunity in case natural pollination fails, hand pollination is an option to maintain reproduction in natural population. In case hand pollination is done, our results suggest later‐flowering individuals should be chosen for pollination, to potentially promote the evolution of a more favorable flowering time for natural fruit production.

In conclusion, our study shows that shifts in phenology triggered by global warming cause more damage via frost than reduced opportunities for pollination via desynchronization with the pollinator. It remains to be seen whether this pattern is a more general one for plants with more specialized pollination systems.

## Author Contributions

**Florian P. Schiestl:** conceptualization (lead), formal analysis (lead), resources (lead), writing – original draft (lead), writing – review and editing (lead). **Beat A. Wartmann:** data curation (equal), investigation (equal). **Ruth Bänziger:** data curation (equal), investigation (equal). **Brigitte Györög‐Kobi:** data curation (equal), investigation (equal). **Klaus Hess:** data curation (equal), methodology (equal). **Jürg Luder:** data curation (equal), methodology (equal). **Edith Merz:** data curation (equal), investigation (equal). **Beat Peter:** data curation (equal), investigation (equal). **Max Reutlinger:** data curation (equal), investigation (equal). **Tobias Richter:** data curation (equal), investigation (equal). **Heinz Senn:** data curation (equal), investigation (equal). **Thomas Ulrich:** data curation (equal), investigation (equal). **Beate Waldeck:** data curation (equal), investigation (equal). **Claudia Wartmann:** data curation (equal), investigation (equal). **Roland Wüest:** data curation (equal), investigation (equal). **Walter Wüest:** data curation (equal), investigation (equal). **Quint Rusman:** investigation (equal).

## Conflicts of Interest

The authors declare no conflicts of interest.

## Supporting information


Appendix S1.

## Data Availability

All data underlying the paper has been uploaded with the manuscript for peer review.
