# Supplementary material for: The Late Orchid Catches the Bee: Frost Damage and Pollination Success in the Face of Global Warming in a European Terrestrial Orchid
Source: Ecol Evol. 2025 Jan 16;15(1):e70729. doi: 10.1002/ece3.70729 (PMC11739451; doi:10.1002/ece3.70729)
Supplement: Supplementary file 1 — Appendix S1. [file ECE3-15-e70729-s001.zip › ece370729-sup-0001-AppendixS1/ece370729-sup-0001-FigureS1.pdf]

```
                           10        20        30        40        50        60        70        80        90       100
                   ....|....|....|....|....|....|....|....|....|....|....|....|....|....|....|....|....|....|....|....|
Leaf_ITS2          GGGGTCGCATGTCAATTTGTCAGAAGACAATTGAATTGGGTTAGATGGGAAATGCACGGCTTTCTCATAACAACCTGATGATGAAGAGGAACAACTACGG
Pollen_ITS2        GGGGTCGCATGTCAATTTGTCAGAAGACAATTGAATTGGGTTAGATGGGAAATGCACGGCTTTCTCATAACAACCTGATGATGAAGAGGAACAACTACGG
Clustal Consensus  ****************************************************************************************************

                          110       120       130       140       150       160       170       180       190       200
                   ....|....|....|....|....|....|....|....|....|....|....|....|....|....|....|....|....|....|....|....|
Leaf_ITS2          CTTTCATCCCACCCATTAATCGATCATTGCCAAGAGAAAATCATCCCGCTCTTCAGCCTGCCACACATGATCGCATGACGGGCCACACTCCGCATCCTAG
Pollen_ITS2        CTTTCATCCCACCCATTAATCGATCATTGCCAAGAGAAAATCATCCCGCTCTTCAGCCTGCCACACATGATCGCATGACGGGCCACACTCCGCATCCTAG
Clustal Consensus  ****************************************************************************************************

                          210       220       230       240       250       260       270       280       290       300
                   ....|....|....|....|....|....|....|....|....|....|....|....|....|....|....|....|....|....|....|....|
Leaf_ITS2          ATAAGAACGCTCGTAGCATCGGGCGTCTTATGGAGCGATTTAATGCTTGACGCCCAGGCGGACGTGCCCTTGGCCAGCTGGCCTCAGGCGCAACTTGCGT
Pollen_ITS2        ATAAGAACGCTCGTAGCATCGGGCGTCTTATGGAGCGATTTAATGCTTGACGCCCAGGCGGACGTGCCCTTGGCCAGCTGGCCTCAGGCGCAACTTGCGT
Clustal Consensus  ****************************************************************************************************

                          310       320       330
                   ....|....|....|....|....|....|....
Leaf_ITS2          TCAAAAACTCGATGGTTCACGGAATTTCTGCAAA
Pollen_ITS2        TCAAAAACTCGATGGTTCACGGAATTTCTGCAAA
Clustal Consensus  **********************************
```

select all 100 sequences selected GenBank Graphics Distance tree of results MSA Viewer

	Description	Scientific Name	Max Score	Total Score	Query Cover	E value	**Per. Ident**	Acc. Len	Accession
☑	Ophrys sphegodes subsp. mammosa voucher MKA32 small subunit ribosomal RNA gene, partial sequence; inter...	Ophrys sphegod...	595	595	100%	2e-165	100.00%	724	KY512505.1
☑	Ophrys cf. sphegodes AG-2017 voucher AG 2700 small subunit ribosomal RNA gene, partial sequence; internal tr...	Ophrys cf. spheg...	595	595	100%	2e-165	100.00%	685	KU931727.1
☑	Ophrys sp. AG-2017 voucher AG 2712 small subunit ribosomal RNA gene, partial sequence; internal transcribed ...	Ophrys sp. AG-2...	595	595	100%	2e-165	100.00%	693	KU931726.1
☑	Ophrys scolopax voucher AG 2740 small subunit ribosomal RNA gene, partial sequence; internal transcribed spa...	Ophrys scolopax	595	595	100%	2e-165	100.00%	691	KU931725.1
☑	Ophrys sp. AG-2017 voucher AG 2599 internal transcribed spacer 1, partial sequence; 5.8S ribosomal RNA gene ...	Ophrys sp. AG-2...	595	595	100%	2e-165	100.00%	645	KU931724.1
☑	Ophrys sp. AG-2017 voucher AG 2583 small subunit ribosomal RNA gene, partial sequence; internal transcribed ...	Ophrys sp. AG-2...	595	595	100%	2e-165	100.00%	694	KU931723.1
☑	Ophrys sphegodes subsp. transhyrcana voucher AG 2702 small subunit ribosomal RNA gene, partial sequence; i...	Ophrys sphegod...	595	595	100%	2e-165	100.00%	694	KU931722.1
☑	Ophrys cf. sphegodes AG-2017 voucher AG 2593 small subunit ribosomal RNA gene, partial sequence; internal tr...	Ophrys cf. spheg...	595	595	100%	2e-165	100.00%	694	KU931719.1
☑	Ophrys cf. sphegodes AG-2017 voucher AG 2582 small subunit ribosomal RNA gene, partial sequence; internal tr...	Ophrys cf. spheg...	595	595	100%	2e-165	100.00%	694	KU931705.1
☑	Ophrys sphegodes voucher AG 2586 small subunit ribosomal RNA gene, partial sequence; internal transcribed s...	Ophrys sphegodes	595	595	100%	2e-165	100.00%	694	KU931703.1
☑	Ophrys oestrifera subsp. phrygia voucher personal collection:Kaan Hurkan:119 small subunit ribosomal RNA gen...	Ophrys oestrifera...	595	595	100%	2e-165	100.00%	693	MH050859.1
☑	Ophrys cretica ITS1, 5.8S rRNA gene and ITS2, specimen voucher Chase O-709 K (RBG Kew, UK)	Ophrys spruneri ...	595	595	100%	2e-165	100.00%	630	AM711820.1
☑	Ophrys balearica ITS1, 5.8S rRNA gene and ITS2, specimen voucher Bateman 257 (RBG Kew, UK)	Ophrys x balearica	595	595	100%	2e-165	100.00%	630	AM711803.1
