# Supplementary material for: The Late Orchid Catches the Bee: Frost Damage and Pollination Success in the Face of Global Warming in a European Terrestrial Orchid
Source: Ecol Evol. 2025 Jan 16;15(1):e70729. doi: 10.1002/ece3.70729 (PMC11739451; doi:10.1002/ece3.70729)
Supplement: Supplementary file 1 — Appendix S1. [file ECE3-15-e70729-s001.zip › ece370729-sup-0001-AppendixS1/sm_0001-Supinfo.docx]

**Supplementary information**

**Table S1** Association between flowering time and frost damage for all populations and years separately. Values in brackets are number of plants included in the analysis for each population. Two populations in 2023 (Erlinsbach, Birmenstorf) had no plants with frost damage and were thus not included in this analysis. For the binary logistic regression analysis, frost damage (no-damage/damage) was used as dependent variable and “day of first flower” as independent variable. Significant coefficients are shown in bold.

| Year | Population | Chi^2^ | df | Coefficient (± s.e.) | P |
| --- | --- | --- | --- | --- | --- |
| 2021 | Erlinsbach (51) | 7.44 | 1 | **0.14±0.05** | **0.006** |
|  | Kloten (135) | 0.01 | 1 | 0.00±0.06 | 0.946 |
|  | Küttingen (65) | 6.04 | 1 | **0.09±0.03** | **0.014** |
|  | Birmenstorf (102) | 2.51 | 1 | 0.13**±**0.08 | 0.113 |
|  | Villigen (158) | 19.97 | 1 | **0.22±0.05** | **0.000** |
|  | Villnachern (73) | 10.95 | 1 | **0.18±0.05** | **0.001** |
| 2022 | Erlinsbach (50) | 2.23 | 1 | 0.13**±**0.08 | 0.135 |
|  | Kloten (215) | 1.90 | 1 | 0.25**±**0.18 | 0.169 |
|  | Küttingen (72) | 9.76 | 1 | **0.15±0.05** | **0.002** |
|  | Birmenstorf (61) | 5.133 | 1 | **0.13±0.06** | **0.023** |
|  | Villigen (190) | 38.46 | 1 | **0.17±0.03** | **0.000** |
|  | Villnachern (68) | 6.66 | 1 | **0.15±0.06** | **0.010** |
| 2023 | Kloten (52) | 5.55 | 1 | **0.46±0.19** | **0.018** |
|  | Küttingen (85) | 13.73 | 1 | **0.35±0.09** | **0.000** |
|  | Villigen (172) | 14.80 | 1 | **0.22±0.06** | **0.000** |
|  | Villnachern (70) | 5.51 | 1 | **0.12±0.05** | **0.019** |

**Table S2** Factors impacting percentage of frost damage in the orchid population, including estimated past and future values. The analysis shows that frost damage has increased since the past (significance of time category, see Figure 4) and the association between number of frost days and mean temperature has changed across the time categories (Figure 4; significant interaction between time-category, number of frost days and mean temperature).

| Source | F | df | P |
| --- | --- | --- | --- |
| Time category | 84.6 | 2 | **0.023** |
| No. frost days April | 11.54 | 1 | **<0.001** |
| Mean temp. April | 33.96 | 1 | **<0.001** |
| No. frost days x mean temp. | 0.63 | 1 | 0.428 |
| No. frost days x mean temp x time category | 7.11 | 2 | **0.001** |

**Table S3** Data of flowering time, frost damage, fruit set, number of flowers and plant size in the study populations and throughout the three years of the study. Mean values are followed by standard deviation.

| **Year** | **Population (number of plants surveyed)** | **Mean day of first flower** | **Percent plants with frost damage** | **Number of plants with fruits** | **Total number of fruits** | **Mean number of fruits** | **Percent individuals with fruit-set** | **Mean number of flowers** | **Mean plant size (cm)** |
| --- | --- | --- | --- | --- | --- | --- | --- | --- | --- |
| 2021 | Erlinsbach (115) | 106.04±12.33 | 27 | 0 | 0 | 0.00±0.00 | 0 | 2.98±1.84 | 18.53±5.00 |
|  | Kloten (249) | 115.14±3.09 | 31 | 8 | 9 | 0.09±0.31 | 3 | 3.82±1.72 | 16.73±5.48 |
|  | Küttigen (123) | 107.11±9.31 | 75 | 1 | 1 | 0.04±0.21 | 1 | 5.44±1.61 | 24.15±6.05 |
|  | Birmentorf (132) | 105.75±8.14 | 4 | 20 | 33 | 0.32±0.73 | 15 | 3.19±1.25 | 17.26±6.01 |
|  | Villigen (247) | 104.25±7.74 | 83 | 0 | 0 | 0.00±0.00 | 0 | 4.59±1.60 | 21.36±7.47 |
|  | Villnachern (77) | 90.59±6.81 | 80 | 3 | 3 | 0.04±0.19 | 4 | 5.34±1.70 | 16.95±5.91 |
| 2022 | Erlinsbach (109) | 102.60±12.01 | 27 | 0 | 0 | 0.00±0.00 | 0 | 3.95±1.95 | 11.11±3.76 |
|  | Kloten (284) | 107.88±4.14 | 1 | 5 | 6 | 0.03±0.19 | 2 | 4.53±1.81 | 18.80±6.24 |
|  | Küttigen (129) | 100.70±6.96 | 84 | 1 | 1 | 0.05±0.22 | 1 | 4.95±1.80 | 21.62±7.76 |
|  | Birmenstorf (132) | 101.26±7.59 | 12 | 13 | 20 | 0.29±0.67 | 10 | 3.57±1.50 | 17.64±5.08 |
|  | Villigen (274) | 99.25±7.17 | 41 | 2 | 2 | 0.02±0.12 | 1 | 4.91±2.25 | 23.24±7.72 |
|  | Villnachern (77) | 84.18±6.87 | 47 | 0 | 0 | 0.00±0.00 | 0 | 5.93±1.90 | 13.10±5.51 |
| 2023 | Erlinsbach (116) | 105.26±10.92 | 0 | 0 | 0 | 0.00±0.00 | 0 | 4.97±2.49 | 24.40±7.78 |
|  | Kloten (84) | 106.77±8.43 | 15 | 4 | 6 | 0.11±0.42 | 5 | 6.20±2.28 | 20.87±11.22 |
|  | Küttigen (165) | 101.25±6.17 | 13 | 4 | 4 | 0.04±0.20 | 2 | 5.38±2.55 | 26.56±8.00 |
|  | Birmenstorf (128) | 100.66±6.29 | 0 | 16 | 21 | 0.19±0.50 | 13 | 3.46±2.98 | 26.99±7.44 |
|  | Villigen (274) | 102.64±6.51 | 14 | 2 | 2 | 0.01±0.11 | 1 | 5.55±2.48 | 27.59±10.13 |
|  | Villnachern (77) | 92.20±5.51 | 47 | 4 | 4 | 0.06±0.23 | 5 | 6.30±1.55 | 24.68±8.10 |
| Overall mean ± s.d. | | 101.90±7.11 | 33.38±29.87 | 4.61±5.93 | 6.22±9.19 | 0.07±0.10 | 3.43±4.6 | 4.73±1.03 | 20.64±4.72 |


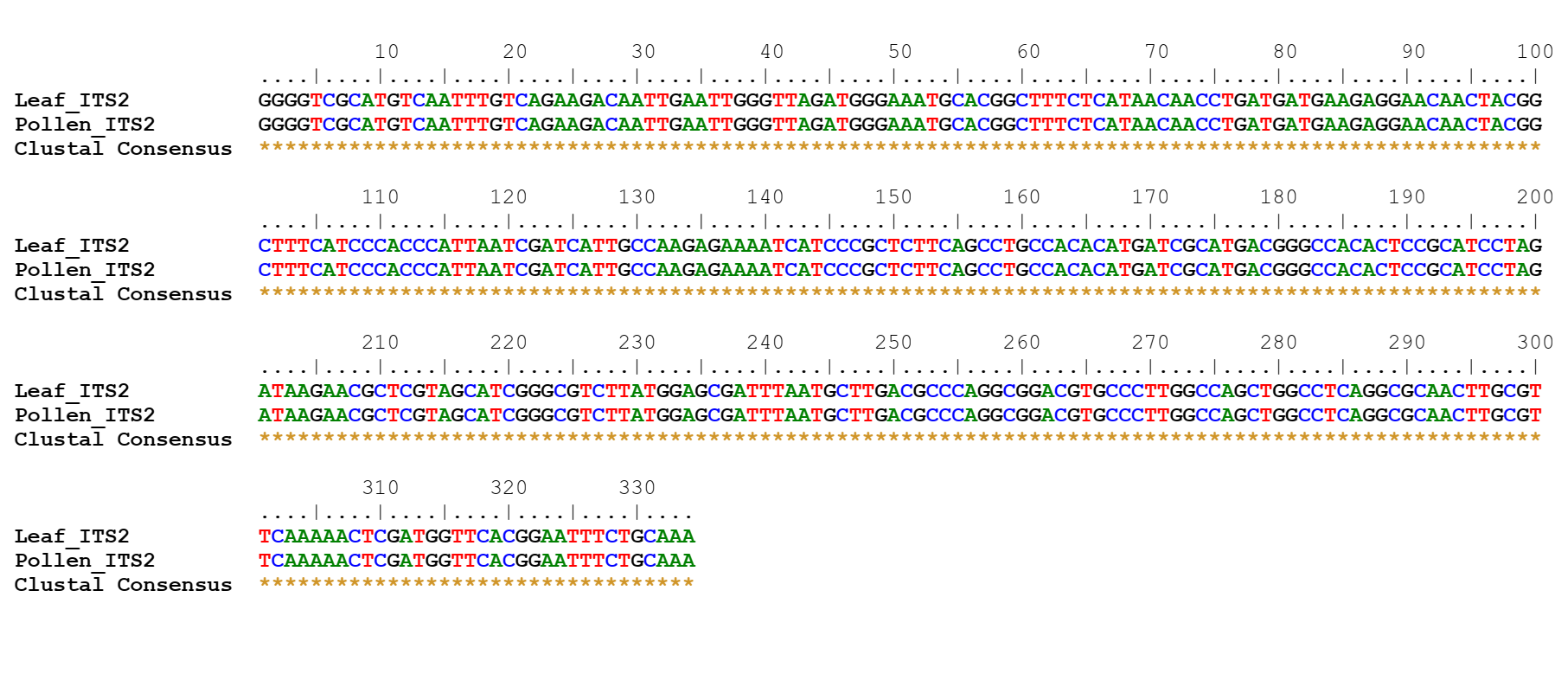


**Figure S1** ITS2 sequences of a pollinarium carried by an *A. combinata* male caught in Birmenstorf, and a leaf sample collected from an *O. araneola* plant in the population.

**Table S4** Association between flowering time and fruit production in the population Birmenstorf for each study year separately. Values in brackets are number of plants included in the analysis for each year. For logistic binary regression, fruit production (no-fruits/fruits) was used as dependent-, and “day of first flower” as independent variable. Significant coefficients are shown in bold.

| Year | Chi2 | df | Coefficient (± s.e.) | P |
| --- | --- | --- | --- | --- |
| 2021 (102) | 15.71 | 1 | **0.19±0.05** | **<0.001** |
| 2022 (64) | 2.81 | 1 | -0.08±0.05 | 0.094 |
| 2023 (82) | 1.23 | 1 | 0.05±0.04 | 0.263 |


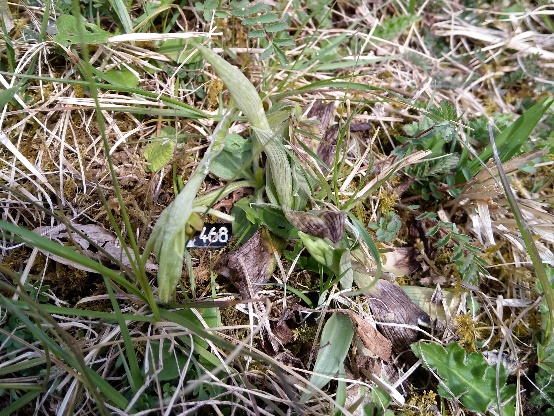

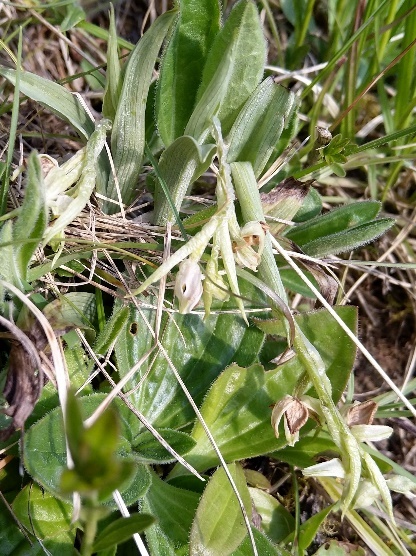

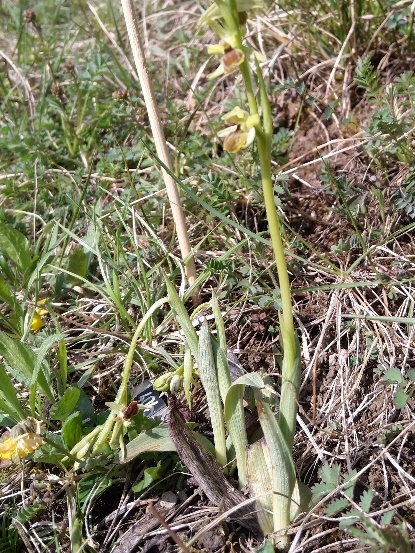


**Figure S2** Pictures of frost-damaged plants in the population Villigen. In the right-hand side picture, a numbering plate is visible. Pictures by Beat Wartmann.
